# Cancer awareness and its related factors among junior high and high school teachers in Japan: a cross-sectional survey

**DOI:** 10.1186/s13690-024-01292-7

**Published:** 2024-05-14

**Authors:** Kumi Suzuki, Naoko Hayashi, Masako Yamanaka, Yoko Minamiguchi, Eiko Yamauchi, Akiko Fukawa, Yasuhiro Tsuda, Yasuhito Fujisaka, Tomoki Doi, Yuko Tomari

**Affiliations:** 1 Faculty of Nursing, Osaka Medical and Pharmaceutical University, 7-6, Hatchonishi-machi, Takastuki, Osaka 569-0095 Japan; 2 Graduate School of Nursing Science, St. Luke’s International University, Chuo-ku, Tokyo, Japan; 3 Faculty of Health Care, Tenri University, Tenri, Nara Japan; 4 Graduate School of Medicine, Ehime University, Toon, Ehime Japan; 5 Faculty of Nursing, Hyogo Medical University, Kobe, Hyogo Japan; 6 Faculty of Medicine, Osaka Medical and Pharmaceutical University, Takatsuki, Osaka Japan; 7 Graduate School of Nursing, Shitennoji University, Habikino, Osaka Japan

**Keywords:** Cancer awareness, Cancer risk factors, Cancer warning signs, Barriers to seeking help, Cancer awareness measure, CAM, High school teachers

## Abstract

**Background:**

The early detection and prevention of many cancers is possible. Therefore, public awareness about cancer risk factors and warning signs must be increased to ensure early diagnosis. Although Japan has implemented mandatory cancer education in junior high and high schools, few studies have evaluated teachers’ cancer awareness. This study aimed to determine Japanese junior high and high school teachers’ awareness of cancer and related factors.

**Methods:**

This cross-sectional study obtained data through an online questionnaire survey using questions from the Cancer Awareness Measure (CAM) developed by Cancer Research UK. Thirty items were selected from three CAM modules: cancer risk factors, cancer warning signs, and barriers to seeking help. Descriptive statistics were used for socio-demografic data and CAM module questions. The χ^2^ test was performed on the relationship between cancer awareness and socio-demographic data. Multiple logistic regression analysis was used to identify factors influencing cancer awareness.

**Results:**

Respondents included 316 junior high school and 463 high school teachers (541 men; 238 women; average age = 48.2 years; average teaching experience = 23.5 years). An average of 5.41 out of 11 cancer risk factors were recognized. More than 70% of teachers recognized smoking, exposure to another person’s cigarette smoke, and having a close relative with cancer as risk factors. On average, 4.52 out of 9 cancer warning signs were recognized. More than 50% of teachers recognized the warning signs of unexplained lump or swelling, unexplained weight loss, and unexplained bleeding. Barriers to seeking help had a low average score of 4.51 out of 20. However, the most commonly recognized “barriers to seeking help” were “too busy to make time,” “difficult to make an appointment,” “worried about what the doctor might find,” and “too scared.” Moreover, the common factors that affected awareness of cancer risk factors and cancer warning signs were gender and cancer experience of relatives. Factors that affected awareness of “barriers to seeking help” were “participation in cancer-related workshops,” age, gender, and cancer experience of relatives.

**Conclusions:**

Cancer awareness education should consider interventions that can improve knowledge of the symptoms and signs related to cancer without increasing the awareness of barriers to seeking help.

**Supplementary Information:**

The online version contains supplementary material available at 10.1186/s13690-024-01292-7.

**Table:** 

**Text box 1. Contributions to the literature**
• This study is the first to use the Cancer Awareness Measure developed by Cancer Research UK to determine cancer awareness and its associated factors among junior high and high school teachers in Japan.
• Japanese teachers’ low awareness of barriers to seeking help should be evaluated.
• The common factors influencing teachers’ awareness of cancer risk factors and warning signs were that they were women, had relatives with cancer, and a high awareness level. Additionally, participation in cancer-related workshops, age, gender and cancer experience of relatives influenced teachers’ awareness of the barriers to seeking help.

## Background

Cancer has become a major global health problem. Data from GLOBOCAN 2020 indicated 19.3 million new cancer cases and almost 10 million deaths from cancer in 2020 [1]. Cancer has become the leading cause of death in Japan, where the incidence rate increases yearly [2]. Japanese people aged < 69 years experienced a high risk of cancer incidence in the colon, stomach, lungs, and prostate among men, and in the breasts, colon, and uterus among women; these sites also present a high risk of mortality [2]. Since some cancers are caused by tobacco, viruses, bacteria, unhealthy diet, lack of exercise, etc. [3], prevention and early detection are possible [4]. Lack of knowledge and awareness about cancer, however, prevents people from receiving early screening [5, 6], affects the timing of visits to medical facilities [7], and contributes to delays in cancer diagnosis [8]. Therefore, people’s awareness of cancer risk factors and warning signs must be increased. In Japan, cancer is the leading cause of death, and it has been pointed out that education to deepen the understanding of cancer itself and to correct the awareness of cancer patients is insufficient [9]. Therefore, it is necessary to develop an interest in health, accurate understanding, and the ability to adopt appropriate attitude and behavior towards cancer by learning about cancer through school education [9].

In Japan, articles on cancer education were newly included in the Cancer Control Act revised in 2016, and awareness and dissemination of cancer education were clearly positioned in the Third Basic Plan to Promote Cancer Control [10]. Subsequently, in Japan, the curriculum guidance for junior high (2017) and high schools (2018) were revised, and cancer prevention and appropriate lifestyle habits were specified in the prevention of lifestyle-related diseases. Since 2021, cancer education has been compulsory in Japanese junior high and high schools [11, 12]. Cancer education in Japanese schools aims to teach about health and the importance of one’s own life and that of others and develop the qualities and capabilities that contribute to creating a society where everyone can live together. This is achieved by deepening an accurate understanding of cancer and empathic understanding of people confronting cancer, including cancer patients and their families [9]. In cancer education in junior high and high schools, the health and physical education classes focus on cancer prevention and recovery, but it is necessary to position and promote health education throughout all education in the school, including special activities and moral studies [13]. In a survey conducted in 2021 on the current state of cancer education for junior high and high schools in Japan [14], 10.6% of junior high schools and 7.1% of high schools invited visiting lecturers to provide cancer instruction. In addition, subjects that tackled cancer were “health and physical education” in 51.3% of junior high schools and 31.6% of high schools, “comprehensive study time” in 27.0% of junior high schools and 13.8% of high schools, and “special activities” in 21.0% of junior high schools and 50.0% of high schools. In other words, 90% of junior high and high school teachers provide cancer education, and it is highly likely that aside from health and physical education teachers and school nurses, many other teachers are also involved in cancer education. Therefore, in order to promote cancer education in junior high and high schools, it is important to raise cancer awareness among teachers. However, few cancer awareness surveys have been conducted among junior high school and high school teachers nationwide.

Accordingly, the aim of this study was to determine the awareness of cancer and its related factors among Japanese junior high and high school teachers.

## Methods

### Study design

This cross-sectional study was conducted in Japan.

### Setting and sample

The subjects were teachers at junior high and high schools nationwide who were registered as monitors with the Internet survey company NEO Marketing, Inc. (hereafter referred to as NEO Marketing). NEO Marketing is a trusted and certified company that has obtained the Privacy Mark.

The selection criteria were as follows: (1) full-time teachers at a junior high or high school, (2) registered as monitors with an Internet survey company NEO Marketing, and (3) consented to cooperate in this study. The exclusion criteria were (1) part-time teachers and (2) teachers working in kindergartens, elementary schools, or universities.

As the response ratio with the largest error was 0.5, we calculated the sample size required for this survey using the equation *n* = λ^2^p (1-p)/d^2^ with a response rate of 0.5, standard error of 5%, and confidence level of 95% (λ = 1.96) [15]. As a result, the sample size was 384 people, and assuming that the valid response rate and awareness of cancer among junior high and high school teachers would be analyzed separately, the number of participants was set at 800, with 400 teachers each.

For sampling, a quota sampling method based on the Ministry of Education, Culture, Sports, Science and Technology Statistical Abstract (2021 edition) [16, 17] was used to ensure equal composition ratios related to gender, age, and regions for junior high and high school teachers nationwide. The gender composition of junior high and high school teachers nationwide was designed such that 60% were men, and 40% were women, with 10% being in their 20s, 25% in their 30s, 25% in their 40s, 35% in their 50s, and 5% in their 60s and over. School locations were sampled so that 13% were in the Hokkaido/Tohoku region, 29% in the Kanto region, 17% in the Chubu (Hokuriku/Tokai) region, 18% in the Kansai region, 10% in the Chugoku/Shikoku region, and 13% in the Kyushu/Okinawa region.

After conducting a screening survey based on selection criteria among junior high or high school teachers from the registered information in NEO Marketing, in the second stage of the study, the survey was conducted using quota sampling based on gender, age, and school location until the target number of participants was met.

### Data collection

Data were collected through an anonymous questionnaire survey via the Internet in December 2021. Sampling and data collection continued until the target number of 800 was met; the questionnaire survey was closed when the target samples were collected.

### Survey instruments

#### Cancer awareness

For questions about cancer awareness, we used the Cancer Awareness Measure (CAM) developed by Cancer Research UK to survey cancer awareness among the public and children [18]. CAM comprises 47 items in nine modules; each module’s selection and use are permitted. This study selected 30 items: (1) Module 2: 9 cancer warning signs; and (2) Module 4: 10 barriers to seeking help (four emotional barriers, three practical barriers, and three service barriers); (3) Module 6: 11 cancer risk factors. These items were translated into Japanese for use in the survey.

To verify the usability and validity of the CAM translated into Japanese, we conducted a discussion among experts in cancer care (two physicians and seven nurses) to determine whether the questions in the CAM were appropriate for Japanese people’s cancer risk factors, cancer warning signs, and barriers to seeking help. Since cancer risk factors differ slightly between Japan and other countries, the experts suggested that the CAM questions should be adapted to address the Japanese risk factors. The opinion was raised that cancer risk factors should be adjusted to the Japanese risk factors. Therefore, we changed “sun exposure” in CAM to “excessive salt intake” with reference to the risk factors proposed by the National Cancer Institute Prevention Research Group [19]. Subsequently, a pre-test of the Japanese version of the CAM was conducted with two junior high or high school teachers. They pointed out that the wording of the five items in the Japanese version of the CAM was difficult to understand. Therefore, the researchers reexamined the text and partially revised the wording of the Japanese version of the CAM so that the meaning of the original text would not be lost.

The response options for cancer risk factors were based on a 5-point Likert scale ranging from “strongly agree” to “strongly disagree”. For cancer risk factors, response options “strongly agree,” and “agree” were defined as 1 and “not sure,” “disagree,” and “strongly disagree” were defined as 0; scaled scores ranged from 0 (not at all aware) to 11 (strongly aware). The response options for cancer warning signs were “yes,” “no,” and “don’t know.” For cancer warning signs, “yes” was taken as 1 and “no” and “not sure” were 0; scaled scores ranged from 0 (not at all aware) to 9 (strongly aware). Higher scores indicated higher cancer awareness. The response options for barriers to seeking help were “often,” “sometimes,” “no,” and “don’t know.” The score for barriers to seeking help was defined as “often”=2, “sometimes”=1, and “never” and “not sure”=0; scaled scores ranged from 0 (do not perceive the barrier) to 20 (strongly perceive the barrier). Higher scores indicated higher awareness of barriers to seeking help.

Internal reliability was confirmed for each module of CAM. Cronbach’s α was 0.79 for risk factors (11 recognition items), 0.77 for warning signs (9 recognition items), and 0.73 for barriers to seeking medical advice (10 recognition items) [20]. The CAM is a face-to-face survey that measures the UK public’s perceptions of cancer symptoms, risk factors, and barriers to seeking help. Online surveys have also been conducted and data comparisons have been made, proving that it is possible to conduct surveys on the Internet [21]. Permission to use CAM was obtained from Cancer Research UK.

#### Socio-demographic data

Socio-demographic data included items such as participants’ gender, age, educational background, marital status, and cancer experience, as well as information about the respondent (self, spouse, family, relatives, close friends). These data were extracted with reference to the demographic questions [18] of the Cancer Awareness Measure developed by Cancer Research UK, and used as individual factors. Factors related to cancer awareness were discussed among the researchers, and the type of school, the entity establishing the school, subjects taught, and the introduction of cancer education at school and participation in cancer-related workshops were set as environmental factors.

### Data analysis

IBM SPSS ver. 29.0 was used for statistical analysis. Descriptive statistics were used for socio-demographic data and questions for each CAM module. Each CAM module’s reliability coefficient was calculated.

Each CAM module’s total score was calculated. Using the median values of cancer risk factors, cancer warning signs, and barriers to seeking help, data were categorized into low awareness and high awareness groups, and univariate analysis (χ^2^ test) was conducted using these awareness items as the dependent variable and individual and environmental factors as independent variables. Next, in order to identify what individual and environmental factors affect awareness of cancer risk factors, cancer warning signs, and barriers to seeking help, multiple logistic regression analysis was performed using the degree of awareness of cancer risk factors, cancer warning signs, and barriers to seeking help as dependent variables, and variables with *p* < 0.1 in univariate analysis were included as covariates. Variables were selected using a variable reduction method using a likelihood ratio test.

The subjects were divided into ages ≥ 40 years (target age for cancer screening; “older group”) and < 40 years (“younger group”). Those who selected “I do not want to answer” in target attributes such as educational background and marital status were excluded. *P*-value < 0.05 was considered statistically significant.

### Ethical consideration

This study was approved by the Osaka Medical and Pharmaceutical University Research Ethics Committee (no. 2021-093). This study was conducted in accordance with the principles of the Declaration of Helsinki. Respondents’ consent was obtained after they received an online explanation of the research purpose, voluntary and anonymous nature of their responses, their anonymity, and that the results would be made public.

## Results

### Sample

Data collection for this survey was terminated when responses were received from 800 teachers, with 779 (97.4%) valid responses. There were 316 (40.6%) junior high school and 463 (59.4%) high school teachers. There were 541 (69.4%) men and 238 (30.6%) women, with an average age of 48.2 years (SD = 10.8) and average teaching experience of 23.5 years (SD = 11.3). In addition, 126 participants (16.2%) responded that cancer education was introduced at school, and only 83 participants (10.7%) responded that they had attended a cancer-related workshop. See Table 1.

**Table 1:** Socio-demographic characteristics of the Japanese junior high and high school teachers participated in the study (*N* = 779; 2022; Japan)

Characteristics	*N*	%
**Gender**		
Women	238	30.6
Men	541	69.4
**Age (years)**		
20–29	49	6.3
30–39	140	18.0
40–49	201	25.8
50–59	279	35.8
≥ 60	110	14.1
**Education level**		
** Bachelor’s degree**	637	81.8
Master’s or doctoral degree	126	16.2
Prefer not to say	16	2.0
**Marital status**		
Married	563	72.3
Single, divorced, or widowed	214	27.5
Prefer not to say	2	0.2
**Cancer experience: self**		
Yes	42	5.4
No	701	90.0
Don’t know	22	2.8
Prefer not to say	14	1.8
**Cancer experience: partner**		
Yes	28	3.6
No	690	88.6
Don’t know	33	4.2
Prefer not to say	28	3.6
**Cancer experience: close family**		
Yes	327	42.0
No	416	53.4
Don’t know	18	2.3
Prefer not to say	18	2.3
**Cancer experience: relatives**		
Yes	284	36.5
No	420	53.9
Don’t know	55	7.1
Prefer not to say	20	2.6
**Cancer experience: close friends or acquaintances**		
Yes	212	27.2
No	463	59.4
Don’t know	86	11.0
Prefer not to say	18	2.3
**School type**		
Junior high school	316	40.6
High school	463	59.4
**School founding group**		
National	19	2.4
Public	587	75.4
Private	173	22.2
**Area of school**		
** Hokkaido and Tohoku**	110	14.1
Kanto	225	28.9
Chubu	134	17.2
Kansai	151	19.4
Chugoku and Shikoku	70	9.0
Kyusyu and Okinawa	89	11.4
**Official title**		
Managerial position	49	6.3
Teacher	692	88.8
Others	38	4.9
**Subjects taught**		
Liberal arts	302	38.8
Science	242	31.1
Arts	31	4.0
Health and sports	80	10.2
Other	124	15.9
**Implementation of cancer education in your school**		
Yes	126	16.2
No	593	76.1
Don’t know	60	7.7
**Participation in cancer-related workshops**		
Yes	83	10.7
No	668	85.7
Don’t know	28	3.6

### Cancer awareness

#### Cancer risk factors

The average number of cancer risk factors was 5.41 of 11 (SD = 3.28). The CAM scale’s reliability coefficient (Cronbach’s α) was cancer risk factors = 0.882. See Table 2.

**Table 2:** Japanese junior high and high school teachers’ scores and reliability coefficients of the cancer awareness measures (*N* = 779; 2022; Japan)

Scale	Score range	Mean	SD	Median	Reliability coefficients (Cronbach’s α)
Cancer risk factors	0–11	5.41	3.28	5.00	0.882
Cancer warning signs and symptoms	0–9	4.52	3.30	4.00	0.899
Barriers to seeking help	0–20	4.51	4.07	4.00	0.875

Over 70% of participants strongly agree or agree with 3 of the 11 cancer risk factors (Table 3). Many participants recognized smoking (78.5%), exposure to another person’s cigarette smoke (71.5%), and having a close relative with cancer (70.6%) as cancer risk factors. Participants agree that other cancer risk factors include being overweight (46.7%), infection with HPV or HCV (45.8%), consuming more than 8 g of salt per day (42.9%), and drinking more than 1 unit of alcohol a day (41.6%). Meanwhile, cancer risk factors for which fewer participants agree were insufficient intake of fruits and vegetables (37.9%), lack of exercise (34.6%), and intake of red and processed meat (30.2%).

**Table 3:** Japanese junior high and high school teachers’ awareness of cancer risk factors (*N* = 779; 2022; Japan) n (%)

Variables	Strongly agree	Agree	Not sure	Disagree	Strongly disagree
Smoking any cigarettes at all	346 (44.4)	266 (34.1)	88 (11.3)	45 (5.8)	34 (4.4)
Exposure to another person’s cigarette smoke	253 (32.5)	304 (39.0)	140 (18.0)	55 (7.1)	27 (3.5)
Drinking more than 1 unit of alcohol a day	91 (11.7)	233 (29.9)	281 (36.1)	127 (16.3)	47 (6.0)
Eating less than 5 portions of fruit and vegetables a day	59 (7.6)	236 (30.3)	302 (38.8)	154 (19.8)	28 (3.6)
Eating red or processed meat once a day or more	56 (7.2)	179 (23.0)	312 (40.1)	179 (23.0)	53 (6.8)
Being overweight (BMI over 25)	95 (12.2)	269 (34.5)	269 (34.5)	115 (14.8)	31 (4.0)
Consuming more than 8 g of salt per day	89 (11.4)	245 (31.5)	319 (40.9)	94 (12.1)	32 (4.1)
Being over 70 years old	78 (10.0)	235 (30.2)	270 (34.7)	136 (17.5)	60 (7.7)
Having a close relative with cancer	159 (20.4)	391 (50.2)	152 (19.5)	59 (7.6)	18 (2.3)
Infection with HPV or HCV	113 (14.5)	244 (31.3)	303 (38.9)	82 (10.5)	37 (4.7)
Engaging in less than 30 min of moderate physical activity 5 times a week	43 (5.5)	227 (29.1)	329 (42.2)	140 (18.0)	40 (5.1)

#### Cancer warning signs

The average number of cancer warning signs was 4.52 of 9 (SD = 3.30). Cronbach’s α of cancer warning signs was 0.899. See Table 2.

More than 50% of participants answered yes to three of the nine cancer warning signs (Table 4). Many participants recognized unexplained lump or swelling (68.2%), unexplained weight loss (61.5%), and unexplained bleeding (56.1%) as cancer warning signs. In contrast, only about 40% of participants recognized the remaining six cancer warning signs.

**Table 4:** Japanese junior high and high school teachers’ awareness of cancer warning signs (*N* = 779; 2022; Japan) n (%)

Variables	Yes	No	Don’t know
Unexplained lump or swelling	531 (68.2)	100 (12.8)	148 (19.0)
Persistent unexplained pain	368 (47.2)	137 (17.6)	274 (35.2)
Unexplained bleeding	437 (56.1)	119 (15.3)	223 (28.6)
Persistent cough or hoarseness	340 (43.6)	163 (20.9)	276 (35.4)
Persistent change in bowel or bladder habits	317 (40.7)	160 (20.5)	302 (38.8)
Persistent difficulty swallowing	344 (44.2)	155 (19.9)	280 (35.9)
Change in the appearance of a mole	375 (48.1)	160 (20.5)	244 (31.3)
Sore that does not heal	331 (42.5)	151 (19.4)	297 (38.1)
Unexplained weight loss	479 (61.5)	115 (14.8)	185 (23.7)

#### Barriers to seeking help

The average score of barriers to seeking help was 4.51 of 20 (SD = 4.07). Cronbach’s α of barriers to seeking help signs was 0.875. See Table 2. Among participants who responded “Yes, often” or “Yes, sometimes” as “Barriers to seeking help,” “being too busy to make time” (68.0%) was the most common response, followed by “difficult to make an appointment” (48.0%), “worried about what the doctor might find” (46.9%), “too scared” (42.0%), and “too many other things to worry about” (39.9%) (Table 5). Fewer than 30% of participants were aware of the other five barriers to seeking help.

**Table 5:** Japanese junior high and high school teachers’ barriers to seeking help (*N* = 779; 2022; Japan) n (%)

Variables		Yes often	Yes Sometimes	No	Don’t know
*Emotional barriers*					
	I would be too embarrassed	39 (5.0)	149 (19.1)	539 (69.2)	52 (6.7)
	I would be too scared	84 (10.8)	243 (31.2)	398 (51.1)	54 (6.9)
	I would be worried about what the doctor might find	97 (12.5)	268 (34.4)	370 (47.5)	44 (5.6)
	I wouldn’t feel confident talking about my symptom with the doctor	31 (4.0)	145 (18.6)	551 (70.7)	52 (6.7)
*Practical barriers*					
	I would be too busy to make time to go to the doctor	238 (30.6)	291 (37.4)	207 (26.6)	43 (5.5)
	I have too many other things to worry about	89 (11.4)	222 (28.5)	401 (51.5)	67 (8.6)
	It would be difficult for me to arrange transport for the doctor’s surgery	32 (4.1)	83 (10.7)	621 (79.7)	43 (5.5)
*Service barriers*					
	I would be worried about wasting the doctor’s time	30 (3.9)	114 (14.6)	593 (76.1)	42 (5.4)
	It would be difficult to make an appointment with my doctor	98 (12.6)	276 (35.4)	340 (43.6)	65 (8.3)
	My doctor would be difficult to talk to	37 (4.7)	171 (22.0)	523 (67.1)	48 (6.2)

### Factors associated with cancer awareness

#### Cancer risk factors

Participants were divided into groups with low and high awareness of cancer risk factors, and a χ^2^ test was conducted with awareness of cancer risk factors as the dependent variable and individual and environmental factors as independent variables (Table 6). There was a significant difference in the group with high awareness of cancer risk factors, with more women (57.1%) than men (42.5%) (χ^2^ = 14.201, *p* < 0.001). Additionally, in the high awareness group, the percentage of individuals below 40 years old (55.6%) who had experienced cancer in their relatives (51.8%) was higher than the percentage of individuals aged 40 years old and above (44.2%) who had such experience (43.6%). This difference was found to be statistically significant (χ^2^ = 7.362, *p* = 0.007; χ^2^ = 4.781, *p* = 0.029).

**Table 6:** Association of Japanese junior high and high school teachers’ socio-demographic characteristics and cancer awareness (*N* = 779; 2022; Japan)

Variable		n (%)	Cancer risk factors					Cancer warning signs					Barrier to seeking help				
			Low Group n= (%)	High Group n= (%)	χ^2^-value	*p*-value	φ coefficient	Low Group n= (%)	High Group n= (%)	χ^2^-value	*p*-value	φ coefficient	Low Group n= (%)	High Group n= (%)	χ^2^-value	*p*-value	φ coefficient
Gender																	
	Women	238 (30.6)	102 (42.9)	136 (57.1)	14.201	< 0.001	-0.135	100 (42.0)	138 (58.0)	9.163	0.002	-0.108	111 (46.6)	127 (53.4)	16.172	< 0.001	-0.144
	Men	541 (69.4)	311 (57.5)	230 (42.5)				291 (53.3)	250 (46.2)				336 (62.1)	205 (37.9)			
Age																	
	20–39	189 (24.3)	84 (44.4)	105 (55.6)	7.362	0.007	-0.097	89 (47.1)	100 (52.9)	0.961	0.327	-0.035	83 (43.9)	106 (56.1)	18.503	< 0.001	-0.154
	40+	590 (75.7)	329 (55.8)	261 (44.2)				302 (51.2)	288 (48.8)				364 (61.7)	226 (38.3)			
**Education level**																	
	Bachelor’s degree	637 (81.8)	338 (53.1)	299 (46.9)	0.217	0.641	0.017	327 (51.3)	310 (48.7)	2.484	0.115	0.057	366 (57.5)	271 (42.5)	0.004	0.948	0.002
	Master’s or doctoral degree	126 (16.2)	64 (50.8)	62 (49.2)				55 (43.7)	71 (56.3)				72 (57.1)	54 (42.9)			
**Marital status**																	
	Single, divorced, or widowed	214 (27.5)	110 (51.4)	104 (48.6)	0.312	0.576	-0.020	103 (48.1)	111 (51.9)	0.502	0.478	-0.025	113 (52.8)	101 (47.2)	2.409	0.121	-0.056
	Married	563 (72.3)	302 (53.6)	261 (46.4)				287 (51.0)	276 (49.0)				332 (53.0)	231 (41.0)			
Cancer experience																	
Self	Yes	42 ( 5.5)	28 (66.7)	14 (33.3)	3.053	0.081	0.063	16 (38.1)	26 (61.9)	2.547	0.111	-0.058	19 (45.2)	23 (54.8)	2.563	0.109	-0.058
	No/Don’t know	723 (94.5)	382 (52.8)	341 (47.2)				367 (50.8)	356 (49.2)				418 (57.8)	305 (42.2)			
Partner	Yes	28 ( 3.7)	17 (60.7)	11 (39.3)	0.649	0.421	0.029	15 (53.6)	13 (46.4)	0.165	0.684	0.015	17 (60.7)	11 (39.3)	0.153	0.696	0.014
	No/Don’t know	723 (96.3)	383 (53.0)	340 (47.0)				359 (49.7)	364 (50.3)				412 (57.0)	311 (43.0)			
Close family	Yes	327 (43.0)	166 (50.8)	161 (49.2)	1.702	0.192	-0.047	142 (43.4)	185 (56.6)	9.718	0.002	-0.113	176 (53.8)	151 (46.2)	2.212	0.137	-0.054
	No/Don’t know	434 (57.0)	241 (55.5)	193 (44.5)				238 (54.8)	196 (45.2)				257 (59.2)	177 (40.8)			
Relatives	Yes	284 (37.4)	137 (48.2)	147 (51.8)	4.781	0.029	-0.079	116 (40.8)	168 (59.2)	15.434	< 0.001	-0.143	148 (52.1)	136 (47.9)	4.760	0.029	-0.079
	No/Don’t know	475 (62.6)	268 (56.4)	207 (43.6)				264 (55.6)	211 (44.4)				286 (60.2)	189 (39.8)			
Close friends or acquaintances	Yes	212 (27.9)	101 (47.6)	111 (52.4)	3.849	0.050	-0.071	97 (45.8)	115 (54.2)	2.053	0.152	-0.052	113 (53.3)	99 (46.7)	1.667	0.197	-0.047
	No/Don’t know	549 (72.1)	305 (55.6)	244 (44.4)				283 (51.5)	266 (48.5)				321 (58.5)	228 (41.5)			
School type																	
	Junior high school	316 (40.6)	161 (50.9)	155 (49.1)	0.912	0.340	-0.034	158 (50.0)	158 (50.0)	0.008	0.929	-0.003	176 (55.7)	140 (44.3)	0.617	0.432	-0.028
	High school	463 (59.4)	252 (54.4)	211 (45.6)				233 (50.3)	230 (49.7)				271 (58.5)	192 (41.5)			
School founding group																	
	National/public	606 (77.8)	315 (52.0)	291 (48.0)	1.177	0.278	-0.039	307 (50.7)	299 (49.3)	0.239	0.625	0.018	350 (57.8)	256 (42.2)	0.157	0.692	0.014
	Private	173 (22.2)	98 (56.6)	75 (43.4)				84 (48.6)	89 (51.4)				97 (56.1)	76 (43.9)			
**Area of school** **Subjects taught**	Hokkaido Kanto Chubu Kansai Chugoku Kyusyu	110 (14.1) 225 (28.9) 134 (17.2) 151 (19.4) 70 ( 9.0) 89 (11.4)	59 (53.6) 120 (53.3) 64 (47.8) 77 (51.0) 40 (57.1) 53 (59.6)	51 (46.4) 105 (46.7) 70 (52.2) 74 (49.0) 30 (42.9) 36 (40.4)	3.764	0.584	0.070	53 (48.2) 115 (51.1) 59 (44.0) 76 (50.3) 42 (60.0) 46 (51.7)	57 (51.8) 110 (48.9) 75 (56.0) 75 (49.7) 28 (40.0) 43 (48.3)	5.063	0.408	0.081	72 (65.5) 127 (56.4) 76 (56.7) 79 (52.3) 48 (68.6) 45 (50.6)	38 (34.5) 98 (43.6) 58 (43.3) 72 (47.7) 22 (31.4) 44 (49.4)	9.896	0.078	0.113
	Health and sports	80 (10.3)	35 (43.8)	45 (56.3)	3.074	0.080	-0.063	40 (50.0)	40 (50.0)	0.001	0.971	-0.001	47 (58.8)	33 (41.3)	0.068	0.794	0.009
	Other	699 (89.7)	378 (54.1)	321 (45.9)				351 (50.2)	348 (49.8)				400 (57.2)	299 (42.8)			
Implementation of cancer education in your school																	
	Yes	126 (16.2)	58 (46.0)	68 (54.0)	2.944	0.086	-0.061	61 (48.4)	65 (51.6)	0.19	0.663	-0.016	62 (49.2)	64 (50.8)	4.108	0.043	-0.073
	No/Don’t know	653 (83.8)	355 (54.4)	298 (45.6)				330 (50.5)	323 (49.5)				385 (59.0)	268 (41.0)			
Participation in cancer-related workshops																	
	Yes	83 (10.7)	38 (45.8)	45 (54.2)	1.951	0.162	-0.050	33 (39.8)	50 (60.2)	4.045	0.044	-0.072	33 (39.8)	50 (60.2)	11.797	0.001	-0.123
	No/Don’t know	696 (89.3)	375 (53.9)	321 (46.1)				358 (51.4)	338 (48.6)				414 (59.5)	282 (40.5)			

There was no association between the awareness of cancer risk factors of teachers and education level, marital status, subjects taught, or area of school. [Table 6]

In the multiple logistic regression analysis results, awareness of cancer risk factors was affected by gender (OR: 1.581, 95% CI: 1.126–2.221, *p* = 0.008), cancer experience of relatives (OR: 1.499, 95% CI: 1.103–2.035, *p* = 0.010), and implementing cancer education in school (OR: 1.602, 95% CI: 1.068–2.404, *p* = 0.023) (Table 7).

**Table 7:** Summary of multiple logistic regression analyses for variables predicting Japanese junior high and high school teachers’ cancer awareness (*N* = 779; 2022; Japan)

Dependent variable	Predictors	OR	95% CI		*p*-value	Model χ^2^ test	Hosmer-Lemeshow test	Approval rate
			Lower	Upper				
Cancer risk factors						*p* < 0.001	0.683	57.4
	Gender	1.581	1.126	2.221	0.008			
	Cancer experience: relatives	1.499	1.103	2.035	0.010			
	Implementing cancer education in school	1.602	1.068	2.404	0.023			
	Age	1.420	0.981	2.056	0.063			
	Cancer experience: self	1.961	0.955	4.024	0.066			
Cancer warning signs						*p* < 0.001	0.674	57.7
	Cancer experience: relatives	1.768	1.308	2.392	< 0.001			
	Gender	1.652	1.201	2.273	0.002			
	Participation in cancer-related workshops	1.620	0.996	2.636	0.052			
Barrier to seeking help						*p* < 0.001	0.738	62.3
	Participation in cancer-related workshops	2.398	1.465	3.924	0.001			
	Age	1.842	1.268	2.675	0.001			
	Gender	1.578	1.122	2.220	0.009			
	Cancer experience: relatives	1.479	1.086	2.014	0.013			

#### Cancer warning signs

In the group with high awareness of cancer warning signs, there were more women (58.0%) than men (46.2%), and a significant difference was observed (χ^2^ = 9.163, *p* = 0.002). Moreover, in the group with high awareness, there were more participants who had cancer experience (family and relatives) (56.6%, 59.2%) than those who did not (45.2%, 44.4%), and a significant difference was observed (χ^2^ = 9.718, *p* = 0.002; χ^2^ = 15.434, *p* < 0.001) (Table 6).

There was no association between the awareness of cancer warning signs of teachers and the level of education, marital status, subjects taught, or area of school.

In the multiple logistic regression analysis results, awareness of cancer warning signs was affected by the cancer experience of relatives (OR: 1.768, 95% CI: 1.308–2.392, *p* < 0.001), and gender (OR: 1.652, 95% CI: 1.201–2.273, *p* = 0.002) (Table 7).

#### Barriers to seeking help awareness

In the group with high awareness of barriers to seeking help, there were more women (53.4%) than men (37.9%), and a significant difference was observed (χ^2^ = 16.172, *p* < 0.001). Moreover, in the group with high awareness, there was a higher percentage of younger individuals (56.1%) compared to older individuals (38.3%) who had experienced cancer in their relatives (47.9%) rather than those who had not (39.8%). This difference was found to be statistically significant (χ^2^ = 18.503, *p* < 0.001; χ^2^ = 4.760, *p* = 0.029). Furthermore, in the group with high awareness of barriers to seeking help, those who responded that they had introduced cancer education (50.8%) compared to those who had not (41.0%) were more likely to have attended a cancer-related workshop (60.2%) than not (40.5%), and a significant difference was observed (χ^2^=4.108, *p* = 0.043; χ^2^ = 11.797, *p* = 0.001) (Table 6).

In the multiple logistic regression analysis results, awareness of barriers to seeking help was affected by participation in cancer-related workshops (OR: 2.398, 95% CI: 1.465–3.924, *p* = 0.001), age (OR: 1.842, 95% CI: 1.268–2.675, *p* = 0.001), gender (OR: 1.578, 95% CI: 1.122–2.220, *p* = 0.009), and cancer experience of relatives (OR: 1.479, 95% CI: 1.086–2.014, *p* = 0.013) (Table 7).

## Discussion

This study was a large-scale cross-sectional survey that examined the cancer awareness of junior high and high school teachers across Japan and the factors associated with that awareness.

Many surveys in Japan have examined awareness of cervical cancer [22] and breast cancer [23] among university students. Moreover, research targeting school teachers includes research on the current status and issues of cancer education [24] and the role of school nurses in cancer education [25]. Considering the recommended introduction of cancer education in junior high and high schools in 2021 and teachers’ role in promoting cancer education, our study surveyed a subject that is critical nationwide.

No prior research in Japan has used CAM [18] developed in the UK. Thus, translating the information into Japanese and conducting a survey using CAM is a significant contribution to clarify Japanese people’s cancer awareness level compared to that of other countries’ citizens. Furthermore, since the reliability coefficients of the Japanese translated CAM scale are high for cancer risk factors, cancer warning signs, and barriers to seeking help (Cronbach’s α = 0.882, 0.889, and 0.875, respectively), the scale’s reliability is confirmed.

### Cancer awareness

Previous studies have shown that male secondary school teachers in Malaysia had very poor knowledge of risk factors related to lung cancer [26], female high school teachers in Saudi Arabia had inadequate knowledge of risk factors and prevention of cervical cancer [27], and about half of primary through high school teachers in Turkey had knowledge of the risk factors and signs of skin cancer [28]. Thus, it was noted that school teachers’ knowledge and awareness of cancer is inadequate in other countries, and the results were similar in Japan. The teachers’ average cancer risk factors number was 5.41 of 11 (SD = 3.28). In a CAM survey, US university students recognized 6.69 of 11 (SD = 3.08) cancer risk factors, on average [29]; thus, Japanese teachers’ awareness of cancer risk factors is lower than that of US university students. The average number for awareness of cancer warning signs was 4.52 of 9 (SD = 3.30). A large-scale survey of US adults using the Awareness and Belief about Cancer (ABC) measure showed a high average awareness of 8.43 of 11 cancer symptoms [30]. The ABC measure includes all nine cancer warning signs of CAM; however, since the total numbers are different, a general comparison cannot be made. Still, Japanese teachers have less awareness of cancer warning signs compared to US adults. Although the importance of cancer education in school has long been recognized [31], in Japan, cancer education was incorporated into the provisions of the 2016 Basic Act on Cancer Control [10], and full-scale implementation has just begun in junior high and high schools from 2021 [11, 12]. Therefore, although teachers are aware of the importance of cancer education for their students, it is believed that they have low knowledge of cancer because they did not have the opportunity to learn about cancer themselves. Therefore, cancer awareness education to raise cancer awareness is necessary for teachers, as they are critical to promote cancer education among junior high and high school students.

Factors that were recognized by many teachers as cancer risk factors were smoking (78.5%) and passive smoking (71.5%). This result was consistent with a survey of Japanese citizens that ranked smoking as the number one cancer risk factor [32]. However, in an online survey of 3,246 adults in the UK aged 18 and above, 98.6% of the respondents from agency A and 95.4% from agency B identified smoking as a risk factor. Similarly, passive smoking was recognized by 88.2% of respondents from agency A and 86.1% from agency B [21]. Hence, it can be said that the awareness of Japanese teachers tends to be low. In Japan, measures against smoking and passive smoking were included in Health Japan 21 in 2000 [33], and the Health Promotion Act regulating passive smoking was revised in 2018 [34]. In this way, although Japan has been implementing long-term anti-smoking measures, it is possible that the knowledge of some teachers has not yet been firmly established.

The most common cancer warning signs recognized by participants were unexplained lump or swelling at 68.2%, unexplained weight loss at 61.5%, and unexplained bleeding at 56.1%. In contrast, in an online survey of adults in UK, unexplained lump or swelling was recognized by 98.4% (samples of agency A) and 94.7% (samples of agency B), unexplained weight loss was recognized by 96.4% (samples of agency A) and 86.5% (samples of agency B), unexplained bleeding was recognized by 89.1% (samples of agency A) and 86.3% (samples of agency B), and other warning signs were also highly recognized by over 70% respondents [21], so awareness among Japanese teachers can be considered low. In Japan, many leaflets have been created to raise awareness and educate about the risk factors for cancer prevention [35, 36], and it is conceivable that leaflets are may be due to the fact that although they are used for awareness activities, there is not much awareness-raising education about cancer warning signs. However, the more people are aware of cancer warning signs, the more likely they are to recognize the barriers to seeking help [37]. Hence, while Japanese teachers need an approach that increases their awareness of cancer warning signs, it is important to pay close attention to avoid increasing the barriers to seeking help.

The average score for awareness of barriers to seeking help was 4.51 of 20 (SD = 4.074); thus, teachers perceive fewer barriers. Japan has a universal health insurance system [38], which means that anyone with health concerns can visit any medical institution at any time. Hence, awareness of barriers to seeking help is low among Japanese people who can freely visit medical institutions as needed. This is a commendable finding. Respondents cited reasons of “too busy to make time” (68.0%), “worried about what the doctor might find” (46.9%) and “too scared” (42.0%) the most as “barriers to seeking help,” among all other reasons. The top reasons why Japanese people do not undergo cancer screening are, in order, as follows: most say that they “don’t have the time,” “have confidence in their health condition and don’t feel it is necessary,” “can always go to a medical institution to be examined when it becomes a worry,” “it will become a financial burden,” and “are scared to find out they have cancer.” [39]. The cancer screening participation rate in 2022 was around 50% for men and less than 50% for women [40]. The results of this survey regarding barriers to seeking help can be considered as useful findings when thinking about approaches to promoting cancer screening behavior.

### Factors associated with cancer awareness

Awareness of cancer risk factors and warning signs was also associated with gender: There were significantly more women than men in the high awareness group. This is consistent with previous studies that found women were more aware of cancer risk factors [29, 32] and cancer symptoms [41]. Since women are at greater risk of cancer due to regular hormonal changes, they are more likely to learn about cancer symptoms [42]. Cancer experiences of relatives is also significantly related to awareness of cancer risk factors and cancer warning signs. This result may be supported by the findings of previous studies, which showed that those with a family history of cancer have a higher awareness of cancer risk factors than those without [41]; they also have a higher level of cancer knowledge [28].

This is probably because people who have experienced cancer through a close relationship have had contact with a cancer patient and are motivated to learn about the disease. However, in this study, the cancer experiences of the participants themselves, those close to them, their spouse, family, relatives and close friends/acquaintances, were analyzed separately, and only a relatives’ experience with cancer affected cancer awareness, which can be considered as a new finding. However, further investigation is needed to understand why only a relative’s cancer experience affects cancer awareness.

No regional disparity was found in teachers’ perception of cancer. It can be inferred that this is due to the promotion of equalization of cancer care in Japan [10] and the introduction of a universal health insurance system [38], which has resulted in equal access to information on cancer care no matter which area an individual is in.

Participation in cancer-related workshops, age, gender, and cancer experience (relatives) affected awareness of barriers to seeking help, and those who attended cancer-related workshops, young people, women, and those with cancer experience (relatives) had higher awareness of the barriers to seeking help. Those who attended cancer-related workshops, young people, women, and those with cancer experience of relatives were more likely to be aware of cancer risk factors and warning signs in the group with high awareness compared to those who did not. Therefore, many participants may have been thinking, “I’m worried that something may be discovered,” or “I’m scared of cancer,” because they understand cancer. This can also be inferred from the finding that awareness of more cancer warning signs was significantly associated with awareness of more barriers to seeking help [37]. High awareness of barriers to seeking help is associated with predicted delays in help-seeking [43]. Hence, when attempting to raise awareness of cancer risk factors and warning signs, the results for barriers to seeking help will need to be closely examined.

## Conclusions

This is the first study to investigate the awareness of cancer and related factors among junior high and high school teachers in Japan using CAM. In this survey, it was found that junior high and high school teachers’ awareness of cancer risk factors and cancer warning signs was low, and that gender and cancer experience (relatives) were factors influencing this. Teachers’ barriers to seeking help were generally low. However, in the group with high awareness of barriers to seeking help, there were more participants in cancer-related workshops, young people, women, and people with experience of cancer in relatives. It is important to deepen our understanding of the related factors revealed by this study and implement educational approaches that lead to increased cancer awareness so that the barriers to seeking help do not become high.

The target population for this study is teachers registered with Internet companies, and sample bias is undeniable. Therefore, it is necessary to conduct a large-scale survey by randomly selecting teachers from middle and high schools across the country.

In order to actively promote cancer education in Japanese junior high and high schools in the future, it is important to study the content and methods of cancer education intervention for teachers.

### Electronic supplementary material

Below is the link to the electronic supplementary material.

Supplementary Material 1

## Data Availability

The datasets used and/or analyzed during the current study are available from the corresponding author on reasonable request.

## Abbreviations

CAM: Cancer Awareness Measure
ABC: Awareness and Belief about Cancer
