# Supplementary material for: Cancer awareness and its related factors among junior high and high school teachers in Japan: a cross-sectional survey
Source: Arch Public Health. 2024 May 14;82:71. doi: 10.1186/s13690-024-01292-7 (PMC11092096; doi:10.1186/s13690-024-01292-7)
Supplement: Supplementary file 1 — Supplementary Material 1 [file 13690_2024_1292_MOESM1_ESM.docx]

**The survey form (Cancer Awareness Measure and sociodemographic questions)**

**Cancer Awareness Measure**

| **1. Do you believe the following are signs of cancer? Please select one that applies.** | | | |
| --- | --- | --- | --- |
|  | Yes | No | Don’t know |
| Do you think an unexplained lump or swelling could be a sign of cancer? | □ | □ | □ |
| Do you think persistent unexplained pain could be a sign of cancer? | □ | □ | □ |
| Do you think unexplained bleeding could be a sign of cancer? | □ | □ | □ |
| Do you think a persistent cough or hoarseness could be a sign of cancer? | □ | □ | □ |
| Do you think a persistent change in bowel or bladder habits could be a sign of cancer? | □ | □ | □ |
| Do you think persistent difficulty swallowing could be a sign of cancer? | □ | □ | □ |
| Do you think a change in the appearance of a mole could be a sign of cancer? | □ | □ | □ |
| Do you think a sore that does not heal could be a sign of cancer? | □ | □ | □ |
| Do you think unexplained weight loss could be a sign of cancer? | □ | □ | □ |

| **2. Sometimes people put off going to see the doctor, even when they have a symptom that they think might be serious. How often do you put off going to the doctor because of the following?** | | | | |
| --- | --- | --- | --- | --- |
|  | Yes often | Yes sometimes | No | Don’t know |
| I would be too embarrassed | □ | □ | □ | □ |
| I would be too scared | □ | □ | □ | □ |
| I would be worried about wasting the doctor’s time | □ | □ | □ | □ |
| My doctor would be difficult to talk to | □ | □ | □ | □ |
| It would be difficult to make an appointment with my doctor | □ | □ | □ | □ |
| I would be too busy to make time to go to the doctor | □ | □ | □ | □ |
| I have too many other things to worry about | □ | □ | □ | □ |
| It would be difficult for me to arrange transport to the doctor’s surgery | □ | □ | □ | □ |
| I would be worried about what the doctor might find | □ | □ | □ | □ |
| I wouldn’t feel confident talking about my symptom with the doctor | □ | □ | □ | □ |

| **3. Do you think the following increase the risk of cancer? Please select one that applies.** | | | | | |
| --- | --- | --- | --- | --- | --- |
|  | Strongly disagree | Disagree | Not sure | Agree | Strongly agree |
| Smoking any cigarettes at all | □ | □ | □ | □ | □ |
| Exposure to another person’s cigarette smoke | □ | □ | □ | □ | □ |
| Drinking more than 1 unit of alcohol a day | □ | □ | □ | □ | □ |
| Eating less than 5 portions of fruit and vegetables a day | □ | □ | □ | □ | □ |
| Eating red or processed meat once a day or more | □ | □ | □ | □ | □ |
| Being overweight (BMI over 25) | □ | □ | □ | □ | □ |
| Consuming more than 8 grams of salt per day | □ | □ | □ | □ | □ |
| Being over 70 years old | □ | □ | □ | □ | □ |
| Having a close relative with cancer | □ | □ | □ | □ | □ |
| Infection with HPV (Human Papillomavirus) or  HCV (Hepatitis C virus) | □ | □ | □ | □ | □ |
| Engaging in less than 30 mins of moderate physical activity 5 times a week | □ | □ | □ | □ | □ |

**Sociodemographic Questions**

| **1. What is your gender?** | | | | | | | | | | |  |
| --- | --- | --- | --- | --- | --- | --- | --- | --- | --- | --- | --- |
|  | □ Male |  | | | |  | | |  |  | |
|  | □ Female |  | | | |  | | |  |  | |
|  |  |  | | | |  | | |  |  | |
| **2. What is your age?** | | （　　 　　　　 　） | | | | | | |  |  | |
|  |  |  | | | |  | | |  |  | |
| **3. What is the highest level of education qualification you have obtained?** | | | | | | | | | | |  |
|  | □ Bachelor’s degree |  | | | |  | | |  |  | |
|  | □ Master’s or doctoral degree | | | | |  | | |  |  | |
|  | □ Prefer not to say |  | | | |  | | |  |  | |
|  |  |  | | | |  | | |  |  | |
| **4. What is your marital status?** | | | | | | | | |  |  | |
|  | □ Married |  | | | |  | | |  |  | |
|  | □ Single, divorced, or widowed | | | | |  | | |  |  | |
|  | □ Prefer not to say |  | | | |  | | |  |  | |
|  |  |  | | | |  | | |  |  | |
| **5. Have you, your family or close friends had cancer?** | | | | | | | | | | |  |
|  |  | Yes | No | Don’t know | Prefer not to say | | | | | |  |
|  | You | □ | □ | □ | □ | | | | | |  |
|  | Partner | □ | □ | □ | □ | | | | | |  |
|  | Close family member | □ | □ | □ | □ | | | | | |  |
|  | Relatives | □ | □ | □ | □ | | | | | |  |
|  | Close friends or  acquaintances | □ | □ | □ | □ | | | | | |  |
|  |  |  | | | |  |  |  | | |  |
| **6. What is your school type?** | | | | | | | | |  |  | |
|  | □ Junior high school |  | | | |  | | |  |  | |
|  | □ High school |  | | | |  | | |  |  | |
|  |  |  | | | |  | | |  |  | |
| **7. What type of the entity establishing is your school?** | | | | | | | | | | |  |
|  | □ National |  | | | |  | | |  |  | |
|  | □ Public |  | | | |  | | |  |  | |
|  | □ Private |  | | | |  | | |  |  | |
|  |  |  | | | |  | | |  |  | |
| **8. What area is your school in?** | | | | | | | | | |  | |
|  | □ Hokkaido/Tohoku | | | | |  | | |  |  | |
|  | □ Kanto |  | | | |  | | |  |  | |
|  | □ Chubu |  | | | |  | | |  |  | |
|  | □ Kansai |  | | | |  | | |  |  | |
|  | □ Chugoku/Shikoku | | | | |  | | |  |  | |
|  | □ Kyushu/Okinawa | | | | |  | | |  |  | |
|  |  |  | | | |  | | |  |  | |
| **9. What is your Official title at your school?** | | | | | | | | | |  | |
|  | □ Managerial position | | | | |  | | |  |  | |
|  | □ Teacher |  | | | |  | | |  |  | |
|  | □ Others |  | | | |  | | |  |  | |
|  |  |  | | | |  | | |  |  | |
| **10. What subjects do you mainly teach?** | | | | | | | | |  |  | |
|  | □ Liberal arts |  | | | |  | | |  |  | |
|  | □ Science |  | | | |  | | |  |  | |
|  | □ Arts |  | | | |  | | |  |  | |
|  | □ Health and sports |  | | | |  | | |  |  | |
|  | □ Other |  | | | |  | | |  |  | |
|  |  |  | | | |  | | |  |  | |
| **11. Has cancer education been introduced in your school?** | | | | | | | | | | |  |
|  | □ Yes |  | | | |  | | |  |  | |
|  | □ No |  | | | |  | | |  |  | |
|  | □ Don’t know |  | | | |  | | |  |  | |
|  |  |  | | | |  | | |  |  | |
| **12. Have you ever participated a cancer-related workshop?** | | | | | | | | | |  | |
|  | □ Yes |  | | | |  | | |  |  | |
|  | □ No |  | | | |  | | |  |  | |
|  | □ Don’t know |  | | | |  | | |  |  | |
